# Creasote in Typhoid Fever

**Published:** 1869-06

**Authors:** 


### Creasote in Typhoid Fever.

M. Pécholier of Montpelier, has recently been conducting a series of interesting researches on the action of creasote in typhoid fever. Conceiving the disease to be one, *totius substantiæ*, depending on certain changes in the blood, caused by the action of an organized ferment, which draws from the blood the materials necessary for its nutrition, and exhales those thrown off by its decomposition, M. Pécholier has been led to employ creasote as an anti-fermentive agent. Sixty patients at the Hôpital St. Eloi were chosen as the subjects of the experiment. Every day a draught containing three drops of creasote, two of essence of lime, ninety grammes of water, and thirty grammes of orange-flower water was administered to the patients. At the same time, enemata were given, containing from three to five drops of creasote. M. Pécholier states, as the result of his experiments, that creasote employed in weak doses, either in draughts, injections, or in the form of vapor, at the outset of typhoid fever, acts powerfully in diminishing the intensity of the disease, and shortening its duration. M. Pécholier adds that the employment of the remedy as a prophylactic agent in schools, garrisons, hospitals, etc., during epidemics, would be of extreme efficacy.—*Lancet.*

